# Anti-Smoking Environment: A Perspective from Murray’s Psychogenic Needs Theory

**DOI:** 10.5539/gjhs.v6n1p99

**Published:** 2013-10-27

**Authors:** Kokku Randheer, Mohammad Almotairi, Haseebullah Abdul Naeem

**Affiliations:** 1 Department of Marketing, College of Business Administration (CBA), King Saud University (KSU), Riyadh, Saudi Arabia

**Keywords:** Murray’s psychogenic needs, anti-smoking, campaigning, counseling, general education

## Abstract

Smoking emerged as a social problem in many nations. Smoking is inflicting injuries to society including addiction, diseases, health damage, and loss of productivity. Individuals, institutions and governments are working to contain the menace of smoking. Many policies, programs and activities are being designed and implemented. To extend a helping hand to fight against smoking this study brought to light the amalgamation of Murray’s psychogenic needs theory with anti-smoking activities to create an effective anti-smoking environment. Conceptual methodology is adopted and five propositions were drafted. This study conclude that anti-smoking activities general education, campaigning, counseling, social welfare, and medical camps when moderated by Murray’s psychogenic needs power, affiliation and achievement can create an effective anti-smoking environment further leading to quitting or reduction in the smoking.

## 1. Introduction

World health organization (www.who.int) 2013 reports that each year approximately 6 million die due to tobacco consumption of which 6,00,000 are dying due to in-direct expose to tobacco smoke. They forecast this number to go up to 8 million by 2030. Anti-tobacco activities should educate smokers about the risks inherit with cigarette smoking and motivate smokers to quit smoking. In its report World health organization (www.who.int) 2013 has highlighted that only few understand specific health risks of smoking. Past studies have also documented that smokers are misinformed about the risk of smoking (Pollay et al., 1996) and methods for quitting smoking, so this makes it imperative for public education campaigns on smoking. Over 1 billion people are smoking worldwide and about 80% are living in low and middle-income countries (www.who.int). The total numbers of smokers across the world are expected to increase each year (Fiore et al., 2004). The major reason for this increase understands that tobacco companies are compelled to target the young and women for materialistic profitability (Chaloupka & Warner, 1999). Pollay et al. (1996) find teenagers to be the target future consumers and in some societies with significant disposable incomes. Companies which focus on brand and consumption of their products to be important target younger segment of the society increasing probability of longer term lock-in. Younger people will take a long time to die or quit, thus increasing the likelihood of continued sales (Pollay et al., 1996). Smoking affected all the nations hence it is a global problem. According to Poland et al. (2005), smoking causes economic and non-economic burden on every country (Chaloupka & Warner, 1999). Many nations are fighting smoking at community level to control further damage to the health of people and those suffering from serious health issues (Siegel, 2002). The objective of this study is to propose a model of anti-smoking activities in conjunction with Murray’s psychogenic needs theory.

The present activities aimed at building anti-smoking environment are effective to some extent while much is needed to be done. The reason for this is large number of smokers including students are not favorable in quitting smoking (Wolburg, 2006). Chain-smoking is a challenge to both addicts and social organizations (Wolburg, 2006; Gorin & Heck, 2004). Large numbers of smokers have expressed their desire to quit (Godin, Valois, Lepage, & Desharnais, 1992). But the success stories are few (Kim & Seo, 2001). Siegel (2002) in his study identified that anti-smoking activities are important component of comprehensive tobacco control program. They are designed to counter pro-tobacco influences and increase pro-health messages throughout a state, region or community. These activities promote smoking cessation as well as decrease the likelihood of initiation (Poland et al., 2005). They have to be planned, designed and executed in combination with other disciplines and theories (Godin et al., 1992). Hence this study after much literature review finalized model from psychology aimed at behavioral aspect addressing the psych of a human being.

**Figure 1:**
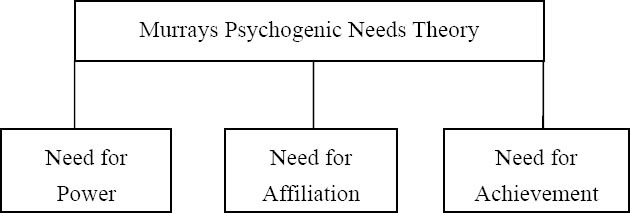
Murray's psychogenic needs theory

Smoking addresses the human psychological need. In this regards the study investigated Murray’s psychogenic needs theory to address anti-smoking activities. Henry Murray (1938) developed psychogenic needs theory between years 1893-1988 for understanding human psychology (refer Figure 1). The theory consisted of three dimensions – one: need for power (nPow), two: need for affiliation (nAff) and three: need for achievement (nAch). Anderson (1988) in his study discussed that these dimensions were based on the human psychological aspects like motives, recognition, belongingness, general needs. Need for power emulates from within an individual to occupy a certain position and professions (supervisors, teachers, parents, elder kids in a family) (Bacharach & Lawler, 1976) which leads to empowering self or creates a desire to dictate, control and influence others (Goodstadt & Hjelle, 1973). The need for affiliation is referred as social need. People identify themselves being a part of a group to exhibit their belongingness or association (Anderson, John, Keltner, & Kring, 2001). The lines are drawn in the mind about groups like religion, race, country, brand community, cause related group, political party and so on (DeWall, Maner, & Rouby, 2009). Need for achievement refers to the inbuilt competitive spirit in the humans (Anderson & Kilduff, 2009) which allows them to gauge their performance or achievement either against self-established standards or standards followed by the others.

Research question of the study: when anti-smoking activities are moderated by Henry Murray’s psychogenic needs, will the integrated effort enhance the anti-smoking environment. Will it further help in bringing down the smoking levels?

## 2. Material Review

Need for power states that individuals acquire power. Those who acquire power exhibit personality which influences others (Anderson & Galinsky, 2006). The need for power shadows an individual's aspiration to communicate, coach, teach or influence others to achieve. A person's needs for power (nPow) are two types: personal and institutional. Those who gain personal power dictate others in achieving what they desire. On the other hand those who gain institutional power apply it to achieve organizational objectives (Burke, 1965). As identified by Burke (1965) persons with need for institutional power are effective than personal power. In other way it can be said that institutional power is more effective and useful than personal power (Bacharach & Lawler, 1976). But there can be an argument saying that one who has personal power if given institutional power can achieve better results (Goodstadt & Hjelle, 1973). For achieving effective anti-smoking environment more institutional power need to be created and operated by a person with high personal referent power. Referent power is derived by individual’s charisma, interpersonal skills, personality and respect.

According to Goodstadt and Hjelle (1973), in the institutional power people with high need for power is perceived to impact others and build their reputation, position and authority. It has been established that people with a high need for power have a more active, assertive and controlling way in their interactions with others (Anderson et al., 2001). The need for power is important because it indicates the individual's desires for influence over others (Anderson & Kilduff, 2009). There are several examples like a teacher who holds referent power at institutional level can be directed or involved in anti-smoking activity to guide his students or influence them against smoking, similarly a manager in a company, a father at home, a priest at worship place.

Need for affiliation has a great impact on personality. The need for affiliation show that those with a high (nAff) have well-built socialization with majority of classes of the society (Anderson et al., 2001). Anderson and Kilduff (2009) in their study discussed that individuals with high in trait dominance influence groups with different ways of behaviour by speaking more, social relations to appear competent or recognized. Their study indicates that persons with high (nAff) interact with others on regular basis, mostly they try to reach mass numbers by using available media. Today with the advent of social media their objective is achieved at lower cost and time. It is learnt that individuals try to affiliate with groups. Hence groups with visible presence have to be developed using religion, education, technology, geographic and on many more factors. Using today’s technology like facebook or twitter one can build groups which have similarity in thoughts towards anti-smoking. This group further can influence other groups (Bacharach & Lawler, 1976).

Need for achievement arises for doing things in one’s own interest. Achievement is referred as “I am doing”, “it's my mission”, “I can do”, “it's my belief” and “I will do it for myself”. Need for achievement (nAch) is directed towards achieving specific goals or performing tasks (Goodstadt & Hjelle, 1973). Though their pursuit for achievement is driven by self-motivation they also try to gain source of motivation from the external environment. Delorme et al. (2003) have discussed that smoking is considered as achievement by people during the initial stages. Smoking starts when an individual is a part of group hence social achievement is felt. This process builds a physiological up- gradation of an individual’s image. Further it will leave its impact on the whole group (Wolburg, 2006). Anti-smoking efforts need to focus on addressing individual’s achievement attitude and also groups.

**Figure 2:**
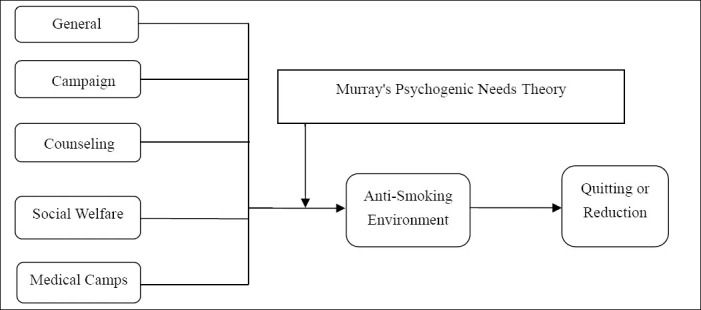
Proposed Model for Quitting Smoking and Anti-Smoking “Murray’s psychogenic needs theory effect on anti-smoking activities”

The proposed model “Murray’s psychogenic needs theory effect on anti-smoking activities” tries to attempt to link Murray’s psychogenic needs - need for power (nPow), need for affiliation (nAff) and need for achievement (nAch) with anti-smoking activities. The model of this study (refer Figure 2) identifies five anti-smoking activities through general education, campaigning, counseling, social welfare, and medical camps to be independent variables. These independent variables are moderated by Murray’s psychogenic needs effecting the dependent variable anti-smoking environment and further leading to smoker’s reducing their smoking intensity or completely quitting it.

### 2.1 General Education

Prevention is better than cure. Educating the smokers or people in general about the side effects of smoking activity before actually they start is a better strategy. It is difficult to know the initial cause influencing smoking activity (DeLorme et al., 2003; Godin et al., 1992; Gaviria & Raphael, 2001) specifically among children (DeLorme et al., 2003). Researchers like DeLorme et al. (2003) argue that it is possible to pen down the reasons behind a person specifically young adult starting smoking. An association can be developed between Murrays' theory with education. Education is an area which can be used effectively to fight against smoking (Ross & Mirowsky, 1999). Gaviria and Raphael (2001) found that education is very effective for the adolescents, especially those who are in high schools. Teachers at school level can achieve the institutional goal of creating anti-smoking effect. Teachers can influence students to a large extent using their personal power in relation to students (Wolburg, 2006). Students have the required acceptability level towards teachers talk and get influenced by them. Teachers can give examples to stress the students on smoking effects. According to the theory X unless external force is not applied people will not act hence teachers are a good option in case of students. Teachers develop affiliation with the students hence their words strike their minds very deep and leave a long-term impact (Ross & Mirowsky, 1999).

Smoking often begins in adolescence, according to Charles et al. (2000) as high as 33% of adult smokers start smoking before the age of 20 hence reducing adolescent smoking is essential. Policy change, specifically curriculum changes are necessary to include anti-smoking campaign into basic education. Apart from efforts done by teachers, at school level different smoking prevention strategies should be initiated like school based educational interventions, community service, advertising at all possible places within the school premises, parent counseling, access restrictions and direct restrictions on smoking.

**Proposition 1:** A person's need for power (nPow) drives him to create an environment in which he exerts pressure or influence while educating (education of) others hence an association can be explained between these two variables further enhancing anti-smoking environment.

Note 1: At College of Business Administration (CBA), King Saud University (KSU), the smoking control committee (SCC) insists faculty members to counsel the students in class against smoking.

### 2.2 Campaigning

Campaigning is an ineffective effort being used to convince smokers to stop is a discouraging fact. On the other hand same is effective in preventing or scaring non-smokers from starting to smoke (Wolburg, 2006). One of the major tools of campaigning is advertising. Goldman, 1988 had identified that positive association observed between exposure to state-sponsored anti-tobacco television advertising and increased rates of quitting among adult smokers supports the need for state governments to continue investing in antismoking advertising. Advertising is complete when based on specific goals. Murrays' theory contributes need for achievement (nAch) to anti-smoking advertising. Three smoking prevention strategies are explored in the literature for teenagers which directly and in-directly aims at need for achievement (nAch) through interventions at school level, mass media/public education and enforcement of cigarette prohibition to minors (Kaplan & Weiler, 1997; Gaviria & Raphael, 2001; Siegel, 2002; Fiore et al., 2004). Wolburg (2006) found that a major effort is advertising at institution level. As students spend 40% of their time in day at institution, aggressive advertising within the school premises can achieve a good result. Like advertising focusing on segregating between smokers and non-smokers, gender (female) rejection of male, social acceptance, effect on class performance, personality failures (Goldman, 1998). Advertising which communicates differently with smokers and non-smokers try new strategies that are more supportive to the effort to quit are far more likely to succeed (Wolburg, 2006). Research shows that there is an increasing trend among the people wishing to quit smoking after getting exposed to campaigns for a continuous long term period (Siegel, 2002). Quitting on this basis is built on various grounds like availability of new successful treatment solutions eliminating nicotine addiction, giving hope for smokers to quit and enhance their image and social belongingness. It will also develop confidence of getting rid of nicotine addiction and its severe withdrawal symptoms.

**Proposition 2:** Need for achievement (nAch) establishes specific targets to be achieved by anti-smoking campaigning through advertising which can directly improve anti-smoking environment.

Note 2: At CBA, KSU advertising is done aggressively and continuous. Fear oriented advertisements and “no-smoking” boards are installed at respective places by SCC.

### 2.3 Counseling

Counseling is convincing people by ways which benefit them (Li, Sun, Liu, Zhang, & Cheng, 2007). It is very difficult to convince people to stop smoking. It’s a tough issue for authorities at institutional level (Li et al., 2007). Considerable size of people smoke at institutional level or at home (Oberga et al., 2010). Also majority of them smoke in public places where there are no restrictions (Poland et al., 2005). Highlighting risks among them who feel invincible may serve to increase the attractiveness of smoking as forbidden fruit (Strecher et al., 1991).

Murray’s Need for affiliation (nAff) can contribute counseling against anti-smoking objective. Murrays’ contended that environmental forces played a significant role in the exhibition of the psychogenic needs. He called the forces "press", indicating a person when thrown into a situation demanding pressure will take him to the boiling point forcing to act. He further argued for a difference between the real environmental forces. Counselors can blend affiliation to convince and motivate people (Gorin & Heck, 2004; Li et al., 2007). Psyche has a deep relation with human behaviour, if associated with counseling moderated by affiliation will be effective.

Every individual needs affiliation to satisfy ego at personal level which is amorphous and cluttered. The belongingness component within an individual’s affiliation needs drives her/him towards groups accepted norms (Gaviria & Raphael, 2001). The focus of counseling should be directed towards addressing group’s behaviour rather than individual (Li et al., 2007). This mean counseling has to influence the group, which results in group behaviour being taken in a particular way which in general is followed by individuals with small amounts of variation in behaviour. Hence this helps individuals to satisfy their ego-needs of being affiliated to group (Anderson & Kilduff, 2009).

**Proposition 3:** Need for affiliation (nAff) for recognition, self-respect and belongingness exerts psychological pressure on individual. Hence counseling directed by need for affiliation creates anti-smoking environment.

### 2.4 Social Welfare

Social welfare activities funded by the government and are executed by its departments and non-government organizations (NGOs) (Kaplan & Weiler, 1997). Their role towards anti-smoking environment is very significant (Siegel, 2002). They are considered to be major contributors against smoking. Welfare by de-addicting smokers using nicotine replacement therapies is effective. David et al. (2009) have found that this method helps smokers to abstain from smoking for sustained time periods. It is also necessary that information related to these activities reach target audiences by various communication platforms like sending flyers, phone calls, e-mails and also by creating social networking groups. Any welfare activity including the one mentioned above can be effective only when their explicit objectives are achieved (Orleans, Rotberg, Quade, & Lees, 1990). Hence welfare institutions have performance accountability and drive on achievement (Fiore et al., 2004). Murray’s need for achievement (nAch) has an association with performance of welfare institutions. Welfare activities in educational institutions are seriously pursued by respective governments in order to prevent new generation from smoking and its related environment (Gaviria & Raphael, 2001). Nick et al. (2011) in their research have discussed for the welfare of the society at large partial ban on smoking is not effective rather creating smoking zones within the establishment premises can help smokers to smoke in the specified area warding of passive smoking. Effective welfare measures help in reaching the goals of anti-smoking (An et al., 2008).

**Proposition 4:** need for achievement (nAch) establishes specific targets to be achieved by anti-smoking campaigning through social welfare activities which can directly improve anti-smoking environment.

Note 3: At CBA, KSU smoking places are allocated with visible boards, and security guards ensure students smoke only in these places

### 2.5 Medical Camps

Medical Camps are important to educate the smokers by one to one interaction (Kim & Seo, 2001). Medical camps educate people on the side effects of smoking, de-motivate smokers by discussing appropriate cases and also provide basic treatment for health related issues (Kaplan & Weiler, 1997). Murray’s need for achievement (nAch) has a role to play in planning and implementation of medical camps hence a proposed variable for anti-smoking. Gorin and Heck (2004) view that medical camp like environment can provide health tips to control oneself internally, provide free of cost services to people. Also convince adults and youth to quit or reduce smoking. In medical camps people are advised on tobacco addiction with a standard framework to prevent and quit. Camps are organized in places like educational institutions, hospitals, public forum, government offices, ministries (An et al., 2008). Feedback from the smokers on usefulness of medical camp will enable to measure the achievements against the goals. The use of Murrays' need of achievement (nAch) should be coined to tailor the training aspect to focus on achievement while planning and executing the medical camps.

**Proposition 5:** Need for achievement (nAch) establishes specific targets to be achieved by anti- smoking campaigning through medical camps which can directly improve anti-smoking environment.

Note 4: At CBA, KSU every semester a medical camp is organized by university hospital which collects blood samples and counsel students against smoking.

## 3. Conclusion

There are two cost facts which result from the implications of smoking. Firstly cost of losing lives and health and the second being the cost of efforts being undertaken by respective governments to fight smoking (Chaloupka, 1999; Poland et al., 2005). Apart from severe effects on smokers there are other direct and indirect implications of smoking on society. The strategy to counter the alarming situation needs a systematic approach by adopting a grand framework which includes different approaches. Governments across the board have initiated campaigns, counseling, social welfare, general education and medical camps to educate and bring awareness among smokers to reduce smoking or discard it. In this direction the study has contributed by trying to locate the anti-smoking activities within the centripetal force of Murray’s psychogenic needs. It means if anti-smoking activities are planned and executed in association with psychogenic needs it will enhance the effectiveness of activities further.

## 4. Societal Implications

This study tried to bridge association between the behavioral theory “Murray’s psychogenic needs” and activities of anti-smoking. Smoking is a social issue and problem (Poland et al., 2005). Smoking creates a host of problems in the society which range from psychological to physiological. Physiological problems are tangible and can be seen, felt by smokers like asthma, cough, chest burning, damage to upper and lower respiratory track, heart disease, lung disease. Smokers affect themselves and when they smoke in public places, at home and at office create second hand smoking environment (SHS). SHS affects the health of women and children at home, colleagues at office and others at public places. Psychologically when smokers become ill due to addiction it pulls their confident levels to low affecting their achievement levels. Further this will create resonance effect on smoker’s affiliation among related people. Hence this study will help understand the issues of achievement, affiliation and power to be included into anti-smoking activities for effectively pitching anti-smoking environment.

## 5. Future Research Studies

This study tried to apply Murray’s psychogenic needs as catalyst within anti-smoking activities to further enhance the effectiveness of creating environment against smoking. The conceptual development was initiated along with relevant propositions. Further to strengthen the study model empirical investigation is needed. Aiming to create affective anti-smoking environment by using mediating variables from Murray may not be sufficient hence it needs additional variables from other theories related to needs and motivation.

## 6. What this Paper Contributes to Literature

The present study investigates anti-smoking activities and efforts by government institutions and private non-profit social organizations in creating effective anti-smoking environment. To enhance further the effectiveness of anti-smoking activities this study identified and added Murray’s psychogenic need theory for evolving a new conceptual model to create anti-smoking environment which controls smoking activity.

Five propositions have been developed and supported by literature. Propositions bring to light, when anti-smoking activities are moderated by Murray’s psychogenic needs they will produce greater effect on anti-smoking environment and further improve the chances for addicts to quit or reduce.
